# NPAS2 Regulation of Anxiety-Like Behavior and GABAA Receptors

**DOI:** 10.3389/fnmol.2017.00360

**Published:** 2017-11-03

**Authors:** Angela R. Ozburn, Joseph Kern, Puja K. Parekh, Ryan W. Logan, Zheng Liu, Edgardo Falcon, Darius Becker-Krail, Kush Purohit, Nicole M. Edgar, Yanhua Huang, Colleen A. McClung

**Affiliations:** ^1^Portland Veterans Affairs Medical Center, Research and Development Service, Portland, OR, United States; ^2^Department of Behavioral Neuroscience, Oregon Health and Science University, Portland, OR, United States; ^3^Department of Psychiatry, University of Pittsburgh Medical Center, Pittsburgh, PA, United States; ^4^National Institute of Neurological Disorders and Stroke, National Institutes of Health, Bethesda, MD, United States

**Keywords:** Npas2, anxiety, GABAA receptors, mouse models, circadian rhythms

## Abstract

Abnormal circadian rhythms and circadian genes are strongly associated with several psychiatric disorders. Neuronal PAS Domain Protein 2 (NPAS2) is a core component of the molecular clock that acts as a transcription factor and is highly expressed in reward- and stress-related brain regions such as the striatum. However, the mechanism by which NPAS2 is involved in mood-related behaviors is still unclear. We measured anxiety-like behaviors in mice with a global null mutation in *Npas2* (Npas2 null mutant mice) and found that Npas2 null mutant mice exhibit less anxiety-like behavior than their wild-type (WT) littermates (in elevated plus maze, light/dark box and open field assay). We assessed the effects of acute or chronic stress on striatal *Npas2* expression, and found that both stressors increased levels of *Npas2*. Moreover, knockdown of *Npas2* in the ventral striatum resulted in a similar reduction of anxiety-like behaviors as seen in the Npas2 null mutant mouse. Additionally, we identified *Gabra* genes as transcriptional targets of NPAS2, found that Npas2 null mutant mice exhibit reduced sensitivity to the GABAa positive allosteric modulator, diazepam and that knockdown of *Npas2* reduced *Gabra1* expression and response to diazepam in the ventral striatum. These results: (1) implicate *Npas2* in the response to stress and the development of anxiety; and (2) provide functional evidence for the regulation of GABAergic neurotransmission by NPAS2 in the ventral striatum.

## Introduction

Psychiatric disorders are among the most devastating diseases and rank among the top factors involved in loss of productivity, quality of life and reduced life span. Clinical and pre-clinical studies provide strong evidence that circadian rhythms and the genes that make up the molecular clock play a key role in the expression of mood-related symptoms in psychiatric disorders (Falcón and McClung, 2009; Karatsoreos, 2014; Landgraf et al., 2014; Logan et al., 2014). In fact, nearly all psychiatric disorders involve some disruption to the normal sleep/wake cycle and this is often one of the criteria used for diagnosis (McClung, 2013).

Circadian rhythms are regulated by a set of transcriptional/translational feedback loops that make up the molecular clock. The core feedback loop consists of transcription factors Circadian Locomotor Output Cycles Kaput (CLOCK), or Neuronal PAS Domain Protein 2 (NPAS2) and Brain and Muscle ARNT like Protein 1 (BMAL1) forming heterodimers, binding to E-box (CACGTG) sequences and positively regulating the transcription of Period (*Per1*, *Per2* and *Per3*) and Cryptochrome (*Cry1* and *Cry2*) genes. PER and CRY proteins are phosphorylated by casein kinase epsilon 1 (CKE1), form homomers or heteromers and translocate to the nucleus where they can inhibit CLOCK:BMAL1 or NPAS2:BMAL1- mediated transcription (Lowrey and Takahashi, 2000; Wang et al., 2007; Mohawk and Takahashi, 2011). While the circadian genes that drive these molecular rhythms are found in the master pacemaker (the suprachiasmatic nucleus), elements of the molecular clock are expressed throughout the brain and periphery.

Studies have revealed that circadian genes and rhythms significantly contribute to mood, anxiety and depression, as well as reward and motivation (Roybal et al., 2007; Ozburn et al., 2012, 2013, 2015; McClung, 2013; Spencer et al., 2013; Logan et al., 2014; Parekh et al., 2015). Abnormal rhythms are strongly associated with psychiatric diseases like seasonal affective disorder, bipolar disorder, major depression and drug addiction (Mukherjee et al., 2010; McClung, 2011; Salgado-Delgado et al., 2011; Hasler et al., 2012; McCarthy and Welsh, 2012; Li et al., 2013; McCarthy et al., 2013). The relationship between circadian genes, sleep and mood is complex. For some, mood symptoms occur prior to sleep disruptions and for others the sleep disruptions occur prior to mood symptoms. It is noteworthy to mention that many of the therapies used to treat these disorders are known to modulate the circadian clock (Bunney and Bunney, 2013). Additionally, single nucleotide polymorphisms (SNP) in a number of circadian genes have been associated with mood disorders (Desan et al., 2000; Benedetti et al., 2004; Nievergelt et al., 2006; Kishi et al., 2008; Kripke et al., 2009; Lavebratt et al., 2010a,b). SNPs in *Npas2* are associated with seasonal affective disorder and major depressive disorder (Partonen et al., 2007; Soria et al., 2010). However, the role of NPAS2 in mood-related behaviors is unclear. Anxiety is a key symptom commonly associated with mood disorders, and is the focus of the studies presented here.

Our present research focuses on determining whether the circadian gene, *Npas2*, is important for the expression of anxiety-like behaviors. *Npas2* is highly expressed in reward- and stress-related brain regions such as the striatum (Garcia et al., 2000). The striatum has dorsal and ventral divisions, both primarily composed of gamma-aminobutyric acid (GABA)-ergic medium spiny neurons (MSNs). The ventral striatum is a significant point of convergence for this circuitry where it receives dopaminergic input from the VTA, as well as glutamatergic inputs from a number of other brain regions (such as the pre-frontal cortex, amygdala and hippocampus). Previously we found that NPAS2 expression in the ventral striatum (nucleus accumbens; NAc) is localized specifically to dopamine receptor 1 (*Drd1*) containing neurons of the so-called “direct” pathway (Ozburn et al., 2015). This circuit is thought to underlie positive or rewarding associations with salient events. In the current studies, we sought to determine the influence of *Npas2* on anxiety-related behavior, the effects of anxiogenic stimuli on *Npas2* expression, and expand our knowledge of the transcriptional targets of NPAS2.

## Materials and Methods

### Animals

NPAS-lacZ (*Npas2* null mutant) and (+/+, wild-type, WT) mice were assayed in a battery of behavioral assays for anxiety-like behavior, as well as in assay of motor coordination in response to diazepam (behavioral studies were performed at the University of Texas Southwestern Medical Center; UTSW). The generation of Npas2 null mutant (−/−) mice is described in Garcia et al. (2000). Mice were obtained from Dr. Steven McKnight (and are now available as strain 005119 from The Jackson Laboratory, Bar Harbor, ME, USA). Npas2 null mutant mice were bred as heterozygotes tested as homozygotes. Male C57BL/6J mice (The Jackson Laboratory, Bar Harbor, ME, USA) were utilized for *Npas2* knockdown (behavioral assays or qPCR), gene expression following acute Forced Swim Stress (FSS) or Unpredictable Chronic Mild Stress (UCMS), Chromatin Immunoprecipitation (ChIP) experiments and electrophysiological experiments (carried out at the University of Pittsburgh Medical Center; UPMC). All mice were housed in a 12:12 light/dark cycle (lights on at 7 am/ZT0, lights off at 7 pm/ZT12) with food and water *ad libitum*. All animal use was conducted in accordance with the National Institute of Health guidelines and approved by the Institutional Animal Care and Use Committees of the University of Pittsburgh and University of Texas Southwestern Medical Center.

### Anxiety-Related Behavioral Testing in Npas2 Null Mutant and WT Mice

We used a battery of behavioral tests to phenotype mice with a null mutation in *Npas2* as compared with their WT littermates. Separate groups of Npas2 null mutant and WT mice were tested in the following behavioral assays (performed at UTSW animal behavioral core during lights on): elevated plus maze, light/dark box (*n* = 16/genotype) and open field. Detailed methods for these behavioral assays are provided in the supplement.

### Determine the Effects of Acute and Chronic Anxiogenic Stimuli on Striatal Npas2 Expression

#### Acute Anxiogenic Stimulus—Forced Swim Stress (FSS)

To determine whether acute stress alters striatal *Npas2* expression, we subjected mice to 3 days of forced swim or control handling and performed quantitative PCR. Male C57BL/6J mice were group housed and exposed to either brief handling or 10 min of FSS (room temperature water, 4 L glass beaker) daily for 3 days (*n* = 5 mice per group). Mice were euthanized 6 h after last swim stress (during lights on). Striatal punches were collected from slices at the time of death, RNA was isolated and processed for qPCR (primer sets used for qPCR, and data analysis are included in the supplement).

#### Chronic Anxiogenic Stimulus—Unpredictable Chronic Mild Stress (UCMS)

To determine whether chronic stress alters NAc *Npas2* expression, we subjected mice to UCMS or control handling and performed qPCR. Our previous findings (Spencer et al., 2013; Logan et al., 2015) have led us to focus more specifically on the ventral aspect of the striatum, referred to as the NAc henceforth. Mice were group housed and exposed to 6 weeks of UCMS or control handling (*n* = 12 mice per group). UCMS treated mice were subjected to a randomized schedule of 1–2 mild stressors per day, 7 days per week. Detailed methods for the UCMS and handling control treatments are provided in the supplement. Immediately after 6 weeks of UCMS or control handling, mice were sacrificed by cervical dislocation and rapid decapitation at ZT4 and ZT16. Whole brains were dissected and flash frozen on dry ice, sectioned on a cryostat at 200 μm, and NAc tissue was collected using a 1 mm core tissue puncher. Detailed methods for sample processing, primer sets used for qPCR, and data analysis are included in the supplement.

### Determine the Effects of NAc Npas2 Knockdown on Anxiety-Like Behaviors

#### Stereotaxic Surgery

Stereotaxic surgery was performed on adult male C57BL/6J mice using validated shRNAs as described in Ozburn et al. (2015). Details are available in the supplement. Bilateral stereotaxic injections of 1 μl of purified high titer AAV2 (1 × 10^12 vg/rm mL^) encoding a scrambled sequence with no known target (scramble shRNA) or *Npas2* shRNA was injected into the NAc (from Bregma: angle 10°, AP +1.5 mm, Lat +1.5, DV −4.4). Both vectors co-expressed GFP.

#### Anxiety-Related Behavioral Testing in Mice Treated with AAV NAc Scramble or Npas2 shRNA

Mice recovered for 4 weeks in their home cage to allow for full viral expression before behavioral testing began. Serial testing began with the least stressful and ended with most stressful, as previously characterized (Tarantino and Bucan, 2000; Spencer et al., 2013). Previous studies indicate that there is little to no effect on behavioral performance between inter-test intervals of 1 week compared with 1–2 days allowing for rapid and semi-high-throughput screening of behavior (Paylor et al., 2006). An inter-test interval of 1–2 days was adopted for this study in line with our previously published work (Roybal et al., 2007; Mukherjee et al., 2010; Spencer et al., 2013). Behavioral assays were carried out at UPMC in the following order: locomotor response to novelty, elevated plus maze, light/dark box and open field. At the completion of behavioral testing, viral injection placement verification was carried out using immunohistochemical methods as described in the supplement (*n* = 10–13/group).

### Determine Whether NPAS2 Has a Role in the Regulation of GABAergic Neurotransmission in the Ventral Striatum

#### Identification of GABAa Subunit Genes as Transcriptional Targets of NPAS2 Via Chromatin Immunoprecipitation (ChIP)

ChIP was carried out as described in Ozburn et al. (2015). Previously we performed ChIP Seq and identified novel DNA binding targets of NPAS2. Here, we performed separate ChIP experiments using an NPAS2 antibody (H20X, Santa Cruz Biotechnology, Santa Cruz, CA, USA) to confirm findings that NPAS2 binds to several genes encoding subunits of the GABAA receptor. As a control, we also incubated samples with Anti-acetyl-Histone H3 or non-immune rabbit IgG (Upstate Millipore, Billerica, MA, USA). Additional methodological details and primer sets used for PCR verification of ChIP Seq results are listed in the supplement.

#### Determine Whether Npas2 Knockdown Alters Expression GABAa Subunits

We next wanted to determine whether NPAS2 mediates expression of GABAa subunits in the NAc. We used viral-mediated RNAi to address this question to minimize developmental compensation that may have occurred in the Npas2 null mutant mouse. Three weeks following viral-mediated knockdown of *Npas2* in the NAc, animals were euthanized at ZT4 and ZT16 (*n* = 6–8/group/time point). To observe localization of GFP expression using an epifluorescence microscope, 30 μm cryostat sections were immediately fixed and dried. Following identification of correct viral injection targeting, 150–300 μm cryostat sections were used for taking punches of NAc tissue, which were later processed for qPCR as described in Supplementary Materials (primer information listed in Supplementary Table S1) and Ozburn et al. (2015).

#### Assay Whether Npas2 Null Mutant Mice Exhibit Altered Responses to the GABAa Positive Allosteric Modulator, Diazepam

Because several GABAa subunits were identified in the NPAS2 ChIP experiment, we next wanted to determine whether mice with a global null mutation in Npas2 (Npas2 null mutant mice) would exhibit reduced sensitivity to the GABAa positive allosteric modulator, diazepam. Npas2 null mutant and WT mice exhibit similar locomotor activity (Ozburn et al., 2015), thus we chose to assay the effects of a moderate dose of diazepam (3 mg/kg) on motor coordination in Npas2 null mutant and WT mice (*n* = 7/genotype; performed at UTSW, see Supplemental Materials for detailed methodology). Although it would be preferential to determine the effects of diazepam, an anxiolytic drug, in a behavioral test that measures anxiety-like behavior, evidence from several laboratories suggests the genetic background (C57BL/6J) of these mice are not responsive to the anxiolytic effects of diazepam in the assays we used (Griebel et al., 2000; Thompson et al., 2015). We considered testing the effects of diazepam in behavioral assays after viral-mediated RNAi targeting *Npas2* in the NAc. We chose not to move forward with this approach since GABAa receptors are found throughout the brain, and diazepam’s anxiolytic actions have not been localized to a particular brain region.

#### Determine Whether NAc Npas2 Knockdown Alters GABAergic Neurotransmission and Response to Diazepam

To determine whether the observed Npas2-dependent changes in GABAa subunit gene expression have functional consequences on GABAergic neurotransmission by NPAS2 in the NAc, we used slice electrophysiology to perform whole cell patch clamp recordings of *Npas2* shRNA or scramble shRNA infected MSNs. We used viral-mediated RNAi to address this question to minimize developmental compensation that may have occurred in the Npas2 null mutant mouse. Additional details are provided in the supplement. Miniature spontaneous inhibitory currents (mIPSC) and stimulus-evoked inhibitory currents (IPSC) were measured, D-APV (50 μM) and NBQX (5 μM) were included to block ionotropic glutamate receptors and TTX (1 μM) was used to prevent action potential generation in mIPSC recordings. After establishing a stable baseline of mIPSCs or IPSCs, 10 μM diazepam solution was bath applied at a consistent flow rate over 10 min. Events from each cell underwent visual screening and scoring was performed blind to treatment. The amplitude and frequency of miniature events were analyzed offline with Clampfit software (Molecular Devices, Sunnyvale, CA, USA). Peak amplitude of evoked IPSCs was measured and averaged across baseline and treatment conditions and within-subjects analysis were conducted.

### Data Analysis and Statistics

Two-way analysis of variance (ANOVA) was performed to analyze: (1) *Npas2* gene expression data after UCMS (treatment × time factors); (2) locomotor activity in response to a novel environment for NAc *Npas2* shRNA and Scramble shRNA treated mice (viral treatment × time); and (3) electrophysiological measurements of mIPSC amplitude, mIPSC frequency and IPSC decay time in NAc MSNs with *Npas2* shRNA or Scramble shRNA treated mice (viral treatment × drug treatment). Student’s *t*-test (two tailed, unpaired) or Mann-Whitney test (for non-normal data distributions) was performed to analyze data from anxiety-like behavioral testing (elevated plus maze, light/dark box and open field assays) in WT or *Npas2* mutant mice, and NAc Npas2 shRNA or Scramble shRNA treated mice. We performed descriptive statistics analyses to identify the mean, median, standard deviation, standard error of the mean, 95% confidence interval, and to test if the values come from a Gaussian distribution (using the Kolmogorov-Smirnov test and Shapiro-Wilk normality test). For a set of values that fail to exhibit a Gaussian (“normal”) distribution, we analyzed the data using the non-parametric Mann-Whitney U test. This test is used to compare differences between two independent groups when the dependent variable is either ordinal or continuous, but not normally distributed. Student’s *t*-test (two tailed, unpaired) was performed to analyze change in IPSC amplitude with diazepam treatment (percent change from baseline) in NAc Npas2 shRNA or Scramble shRNA treated mice. Data was analyzed and graphed using Graphpad Prism 7.00 for Mac OS X (Graphpad Software, La Jolla, CA, USA). Data are presented as mean ± SEM and *p* < 0.05 is considered statistically significant.

## Results

### Anxiety-Related Behavioral Testing in Npas2 Null Mutant and WT Mice

**Figure 1:**
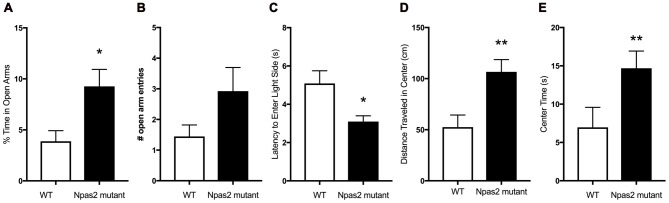
Neuronal PAS Domain Protein 2 (Npas2) null mutant mice exhibited reduced anxiety-like behavior in the elevated plus maze, light/dark box and open filed tests. **(A)** Percent of time spent in the open arms of the elevated plus maze, **(B)** number of open arm entries in the elevated plus maze (*p* = 0.1), **(C)** latency to explore the light side of the light/dark box, **(D)** distance traveled in the center of the open field arena and **(E)** time spent in the center of the open field. Student’s *t*-test or Mann-Whitney U test (see “Results” section): *p < 0.05, **p < 0.01.

In order to determine if functional NPAS2 is important for anxiety-like behavior, we assayed Npas2 null mutant and WT mice littermates in a battery of anxiety-related behavioral tests. We chose to measure their behaviors in the elevated plus maze, light/dark and open field tests to get an overall picture of their anxiety-like phenotype. These tests have been well validated and are replicable in our hands, although outcomes can vary by experimenter, location, apparatus and test, et cetera (Wahlsten et al., 2006; Roybal et al., 2007; Mukherjee et al., 2010 Coque et al., 2011; Arey et al., 2013; Spencer et al., 2013). We previously found that Npas2 null mutant and WT mice exhibit similar locomotor activity (Ozburn et al., 2015). Compared with WT mice, Npas2 null mutant mice exhibited reduced anxiety-like behavior as seen by the increased percent time spent in the open arms of the elevated plus maze (Figure 1A, Mann-Whitney test, Npas2 null mutant median = 7.75, *n* = 24, WT mean = 2.09, *n* = 16, *U* = 115, *p* = 0.032). This was accompanied by an increase in number of open arm entries that was not statistically significant (Figure 1B, Student’s *t*-test, *t*_(20)_ = 1.50, *p* = 0.1). Npas2 null mutant mice showed a reduced latency to explore the light side of the light/dark box (Figure 1C, Mann-Whitney test, Npas2 null mutant median = 2.9, *n* = 16, WT mean = 4.0, *n* = 16, *U* = 70, *p* = 0.028). Lastly, Npas2 null mutant mice exhibited increased distance traveled in the center of the open field (Figure 1D, Student’s *t*-test, *t*_(35)_ = 2.82, *p* < 0.01) and time spent in the center of the open field (Figure 1E, Mann-Whitney test, Npas2 null mutant median = 11.83, *n* = 25, WT mean = 2.67, *n* = 12, *U* = 73, *p* = 0.011). Results from these three different behavioral tests provide the first evidence that a reduction in functional NPAS2 results in reduced anxiety-like behavior.

### Determination of the Effects of Acute and Chronic Anxiogenic Stimuli on Striatal Npas2 Expression

**Figure 2:**
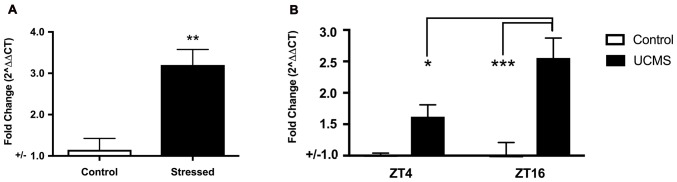
Acute and chronic stress robustly increased Npas2 expression. **(A)** Forced swim stress (FSS) increases striatal Npas2 expression (**p < 0.01), **(B)** unpredictable chronic mild stress (UCMS) results in a robust and significant increase in diurnal nucleus accumbens (NAc) *Npas2* expression as compared with control mice. Tukey’s *post hoc* *p < 0.05, ***p < 0.0001.

As mice with a null mutation in *Npas2* have significant phenotypes in anxiety-related behaviors, we sought to determine whether expression of *Npas2* changes in response to 3 days of FSS or 6 weeks of UCMS. Forced swim test is typically used as a measure of depression-like behavior that is sensitive to antidepressant treatments (Porsolt et al., 1977). A number of studies show that the striatum is important for behavioral responses to stressful stimuli (Kim et al., 2016; Han and Nestler, 2017). Further, it has been shown that a single experience with FSS induces immediate early genes in the ventral striatum (Morello et al., 2017) and that repeated exposure to FSS results in increased time spent immobile (Serchov et al., 2015; Mul et al., 2016). However, here we expose mice to three daily experiences with FSS because it provides a unique paradigm to measure *Npas2* expression in response to stress coping and adaptation (de Kloet and Molendijk, 2016). FSS robustly increased striatal expression of *Npas2* (Figure 2A, Student’s *t*-test, *t*_(8)_ = 4.41, *p* < 0.01). Moving forward, we asked whether chronic stress would also increase *Npas2* expression. We chose to employ UCMS, a paradigm well-known to alter mood-related behaviors, and in our hands reliably induces an increase in anxiety- and depressive-like behavior (Guilloux et al., 2011; Logan et al., 2014). Circadian gene expression in the NAc is responsive to UCMS, where the amplitude of rhythmic expression of *Per2* is increased with UCMS (Logan et al., 2015). We recently found that *Npas2* expression and NPAS2 transcriptional activity in the NAc exhibits diurnal rhythms, with peak levels in the dark and trough levels in the light (when mice are kept in a 12:12 h Light/Dark cycle; Ozburn et al., 2015). Thus, we focused on *Npas2* expression during the peak and trough of the day/night (ZT4 (4 h into the light cycle) and ZT16 (4 h into the dark cycle). We found that UCMS resulted in a robust and significant increase in diurnal *Npas2* expression in the NAc compared to control mice (Figure 2B). Two-way ANOVA revealed a significant group × time interaction (*F*_(1,20)_ = 4.74, *p* < 0.05), main effect of group (*F*_(1,20)_ = 25.18, *p* < 0.0001) and main effect of time (*F*_(1,20)_ = 4.35, *p* = 0.05). Tukey’s *post hoc* analysis revealed that UCMS significantly increased *Npas2* expression at ZT16 (as compared with control, *p* < 0.001) and that mice undergoing UCMS had higher levels of *Npas2* expression at ZT16 (as compared with ZT4, *p* < 0.05). Together, these results suggest that NPAS2 might be involved in the response to acute and chronic stress.

#### Determination of the Effects of NAc Npas2 Knockdown on Anxiety-Like Behaviors

**Figure 3:**
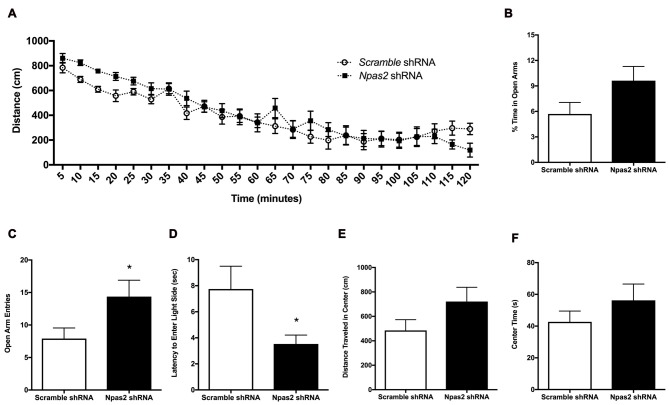
NAc *Npas2* knockdown results in reduced anxiety-like behavior. **(A)** Locomotor response to a novel environment (two-way analysis of variance (ANOVA), treatment × time interaction *F*_(23,805)_ = 2.12, *p* < 0.01; main effect of time (*F*_(23,805)_ = 63.61, *p* < 0.0001), **(B)** % open arm entries in elevated plus maze, **(C)** number of open arm entries in elevated plus maze (*p < 0.05), **(D)** latency to explore the light side of the light/dark box (*p < 0.05), **(E)** distance traveled and **(F)** time spent in the center of the open field arena (*p* = 0.1). *n* = 10–13/treatment.

Because *Npas2* expression in the NAc is highly responsive to the acute and chronic stressors known to be anxiogenic, and the NAc is an important site of neural integration for salient events, we determined if reducing *Npas2* expression in the NAc via viral mediated RNAi (*Npas2* knockdown) was sufficient to reduce anxiety-related behaviors. We first assessed the effects of NAc specific *Npas2* knockdown on locomotor response to a novel environment where analysis revealed a significant treatment × time interaction (Figure 3A, two-way ANOVA, *F*_(23,805)_ = 2.12, *p* < 0.01) and main effect of time (*F*_(23,805)_ = 63.61, *p* < 0.0001). These results reveal that NAc *Npas2* knockdown did not have the same effect over the times measured, suggestive of altered habituation over time. We note that this effect is modest, with no significant *post hoc* results to report, and was not previously observed in Npas2 null mutant mice or in mice with NAc *Npas2* knockdown (Garcia et al., 2000; Ozburn et al., 2015). *Npas2* knockdown in the NAc resulted in an increase in % time in the open arms of the EPM that was not statistically significant (Figure 3B, Student’s *t*-test, *t*_(19)_ = 1.8, *p* = 0.09), and a significant increase in the number of open arm entries (Figure 3C, Student’s *t*-test, *t*_(20)_ = 2.10, *p* < 0.05). In the light/dark box, NAc *Npas2* knockdown reduced latency to explore the light side (Figure 3D, *t*_(20)_ = 2.24, *p* < 0.05). The effects of NAc *Npas2* knockdown were less robust on behaviors in the open field test. *Npas2* knockdown resulted in modest increase in the distance traveled in the center of the open field arena that was not statistically significant (Figure 3E, *t*_(21)_ = 1.59, *p* = 0.1) and did not have an effect on the amount of time spent in the center (Figure 3F, *t*_(21)_ = 1.08, *p* = 0.3). Taken together, NAc *Npas2* shRNA treated mice tended to show reduced anxiety-like behavior (as compared with scramble shRNA treated mice), suggesting that *Npas2* in the NAc contributes to the regulation of anxiety-like behavior.

### A Role for NPAS2 in the Regulation of GABAergic Neurotransmission in the Ventral Striatum

NPAS2 is a transcription factor, and in a previous study we performed ChIP Seq on striatal tissue to identify DNA sequences bound to NPAS2 and identified many novel gene targets, including GABAA alpha (1, 2, 3, 4 and 5), beta (1, 2, 3), gamma (1, 2, 3), epsilon and pi subunits (Ozburn et al., 2015). Here we replicated the findings using ChIP-PCR to seek additional validation and found that indeed NPAS2 binds genes encoding the GABAA subunits alpha 1, 2, 4 and 5, beta 2 and 3, and gamma 1 (Supplemental Figure S1).

**Figure 4:**
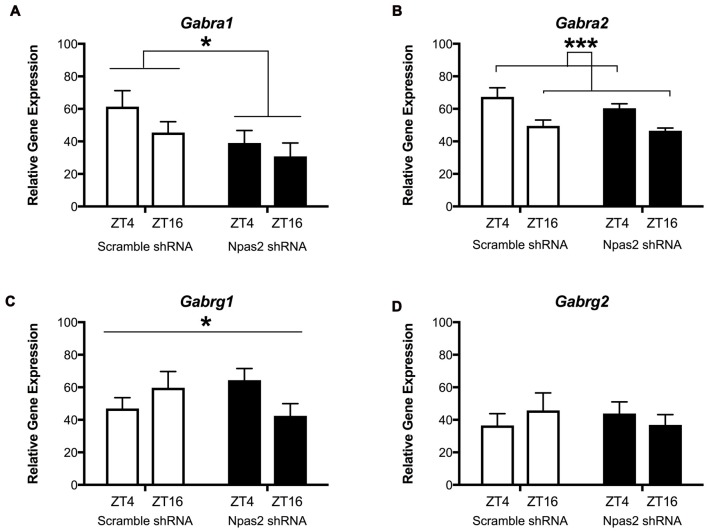
Effect of *Npas2* knockdown on diurnal expression of GABAa subunits in the NAc. **(A)** *Gabra1*–significant effect of knockdown *F*_(1,24)_ = 4.81, *p < 0.05, **(B)** *Gabra2*: main effect of ZT, *F*_(1,25)_ = 15.96, ***p < 0.001, **(C)** *Gabrg1*: significant knockdown × ZT interaction, *F*_(1,25)_ = 4.5, *p < 0.05, **(D)** *Gabrg2*: n/s (as expected).

We next wanted to determine whether NPAS2 mediates expression of GABAA subunits that could indicate synaptic or extra-synaptic receptor localization (*Gabra1, Gabra2, Gabrg1* and* Gabrg2*). Following viral-mediated knockdown of *Npas2* in the NAc, expression of GABAa subunits genes at two time points (ZT16 (lights off) and ZT4 (lights on)) were measured via qPCR. Interestingly, *Npas2* knockdown significantly reduced *Gabra1* expression at both time points measured, suggesting NPAS2 mediates positive regulation of *Gabra1* transcription (Figure 4A; two way ANOVA—main effect of knockdown, *F*_(1,24)_ = 4.81, *p* < 0.05). *Gabra2* exhibited diurnal expression, but was not changed with knockdown (Figure 4B; two way ANOVA—main effect of ZT, *F*_(1,25)_ = 15.96, *p* < 0.01). Intriguingly, the effect of *Npas2* knockdown on *Gabrg1* expression had different effects depending on time of day, suggesting additional factors play a role in its transcriptional regulation (Figure 4C; two way ANOVA—knockdown × ZT interaction, *F*_(1,25)_ = 4.5, *p* < 0.05). Lastly, *Gabrg2* expression was unaltered with *Npas2* knockdown, as expected since it was not identified via CHiP Seq (Figure 4D). These results also suggest that *Gabrg2* expression is not changed in response to changes in other subunits (i.e., there is no compensatory change).

**Figure 5:**
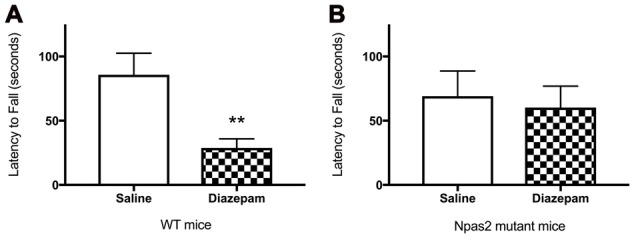
Wild-type (WT) **(A)**, but not *Npas2* mutant **(B)**, mice are sensitive to the motor incoordinating effects of diazepam (3 mg/kg) as determined by the rotorod test (Student’s *t*-test, WT saline vs. diazepam *t* = 3.134, **p < 0.01; *Npas2* mutant saline vs. diazepam *t* = 0.3451, n/s).

To determine whether the absence of functional NPAS2 altered response to a GABAa positive allosteric modulator, we measured motor coordination after administration of diazepam. We observed that WT, but not *Npas2* mutant, mice are sensitive to the motor incoordinating effects of diazepam (3 mg/kg) as determined by the rotorod test (Figures 5A,B; Student’s *t*-test, WT saline vs. diazepam *t* = 3.134, *p* < 0.01; *Npas2* mutant saline vs. diazepam *t* = 0.3451, n/s). These results suggest that mice lacking functional NPAS2 may have altered GABAa subunit composition (changes in expression of diazepam sensitive GABAa receptor subunits) and possibly altered inhibitory transmission.

### Determine Whether NAc Npas2 Knockdown Alters GABAergic Neurotransmission and Cellular Response to Diazepam

**Figure 6:**
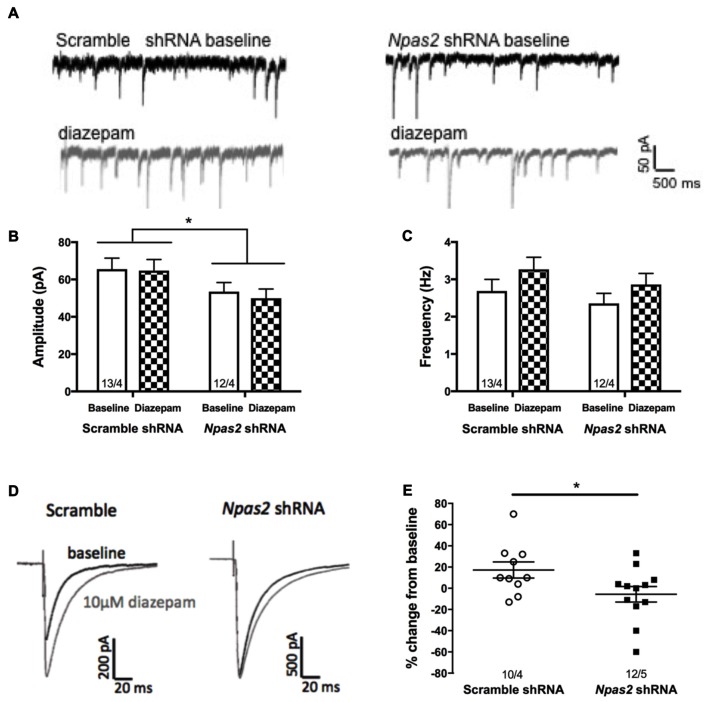
*Npas2* knockdown prevents diazepam-induced potentiation of IPSC amplitude in -NAc medium spiny neurons (MSNs). **(A)** Representative traces of baseline and diazepam mIPSCs from AAV-scramble (left) and -*Npas2* shRNA (right) cells, **(B)** mIPSC amplitude (two way ANOVA, *F*_(1,46)_ = 6.26, *p < 0.05; inset numbers represent number of cells and number of mice, i.e., 13/4 = 13 cells from 4 mice), **(C)** mIPSC frequency (n/s), **(D)** representative traces of evoked IPSC at baseline and following 10 min of diazepam from AAV-scramble and -*Npas2* shRNA infected cells, and **(E)** peak amplitude of the evoked IPSC at baseline and following 10 min of diazepam bath application (10 mM) in scramble and *Npas2* shRNA treated MSNs (Student’s *t*-test, *t* = 2.152, *p < 0.05). *n* = 10–13 cells/group.

In order to assess the effect of *Npas2* knockdown on inhibitory synaptic activity of MSNs, we performed whole-cell patch-clamp recordings in NAc-containing brain slices. We recorded baseline inhibitory miniature currents (mIPSCs) from scramble shRNA and *Npas2* shRNA infected cells to determine effects of knockdown on current amplitude and frequency (indicating alterations in postsynaptic and presynaptic mechanisms, respectively; representative trace shown in Figure 6A). Analysis of mIPSC amplitude revealed a significant main effect of viral treatment (Figure 6B; two way ANOVA, *F*_(1,46)_ = 6.26, *p* < 0.05) but no main effect of diazepam and no significant interaction. Analysis of mIPSC frequency revealed only an increase with diazepam that was not statistically significant (*F*_(1,46)_ = 3.29, *p* = 0.07), and no significant shRNA treatment by diazepam interaction and no main effect of shRNA. These results suggest that *Npas2* knockdown in the NAc alters postsynaptic responses but not presynaptic release of GABA onto MSNs (Figure 6C). However, because we found that mice lacking a functional *Npas2* gene display behavioral insensitivity to diazepam (Figure 5B) and NPAS2 binds to genes encoding GABAA subunits important for pharmacological actions of diazepam (Supplemental Figure S1), we tested whether this insensitivity could be identified at the cellular level. GABAa subunit levels were not assayed using a Western Blot approach due to the lack of specific antibodies for these subunits. Using a combined electrophysiological and pharmacological approach requires functional receptors in cell membranes and provides the most relevant approach to address whether knocking down *Npas2* expression alters GABAergic neurotransmission. Therefore, we measured the peak amplitude of the evoked IPSC at baseline and following 10 min of diazepam bath application (10 μM) in both scramble shRNA and *Npas2* shRNA treated MSNs. We found that diazepam application reliably increased the average IPSC peak amplitude in scramble control cells by approximately 20% while this increase in current amplitude was noticeably absent in *Npas2* shRNA infected cells (Figures 6D,E; Student’s *t*-test, *t* = 2.152, *p* < 0.05; *n* = 10 scramble shRNA, *n* = 12 *Npas2* shRNA). To account for variations in stimulus intensity that inevitably alter the absolute amplitude of IPSCs, we made within-subjects comparisons of baseline and treatment conditions. Additionally, we measured the decay kinetics of IPSCs from both groups under baseline and diazepam conditions. We found that there is a significant main effect of diazepam to prolong the time to half decay of the GABAA receptor-mediated IPSCs (two way ANOVA, *F*_(1,34)_ = 4.90, *p* < 0.05). However, it does so to a similar extent in both scramble- and *Npas2* shRNA treated cells (decay time constants in ms for scramble shRNA: baseline 13.8 ± 1.7, diazepam 19.4 ± 2.5; decay time constants for *Npas2* shRNA: baseline 16.8 ± 3.4, diazepam 24.6 ± 5.0; *n* = 10 baseline scramble shRNA, *n* = 13 baseline *Npas2* shRNA, *n* = 7 diazepam scramble shRNA, *n* = 12 diazepam *Npas2* shRNA), suggesting that NPAS2 specifically modulates diazepam sensitivity of evoked IPSC amplitude in MSNs.

## Discussion

Circadian rhythms and the genes that make up the molecular clock play an important role in the expression of mood-related symptoms in psychiatric disorders. Here we link *Npas2* to responses to stressful and anxiogenic stimuli, expression of anxiety-like behavior, regulation of specific GABAa subunit expression, and inhibitory neurotransmission in the nucleus accumbens.

To determine if *Npas2* is important for the expression of anxiety-like behaviors, we subjected mice lacking functional *Npas2* to a battery of behavioral tests. *Npas2* mutant mice exhibited decreased anxiety-like behaviors as compared with WT mice. *Npas2* mutants exhibited an increased percent time in open arms of the elevated plus maze, reduced latency to explore the light side of the light/dark box, and an increase in the distance traveled in the center of the open field arena. Further, *Npas2* mutant mice were resistant to the motor incoordinating effects of diazepam, suggesting these mice may have altered GABAA receptor subunit composition and reduced synaptic localization. This phenotype is similar to that of *Clock*Δ19 mutant mice, which exhibit reduced anxiety-like behaviors and is the opposite of the *Per1*^−/−^*/Per2*^−/−^ double mutant, which exhibit increased anxiety-like behavior (Roybal et al., 2007; Spencer et al., 2013). Because *Per1* and *Per2* negatively regulate NPAS2/BMAL1 transcription, this further supports a role for NPAS2 in the regulation of anxiety-like behaviors. WT and *Npas2* mutant mice were evaluated in tests of sucrose preference and locomotor response to novelty, with no differences observed (Ozburn et al., 2015). It is interesting to note that although CLOCK and NPAS2 are homologous transcription factors, these mutant mice exhibit opposing drug reward phenotypes. They exhibit brain region and cell-type specific expression differences, as well as many unique transcriptional targets (Ozburn et al., 2015). For example, *Npas2* is highly expressed in limbic and forebrain regions (Garcia et al., 2000). Furthermore, *Npas2* expression has been shown to be specific to dopamine receptor 1 (D1) containing MSNs in the NAc, which are thought to underlie positive or rewarding associations with salient events (Ozburn et al., 2015).

Acute and chronic stress resulted in a robust increase in levels of *Npas2* expression in the NAc. Individuals with mood disorders experience perturbations in a myriad of rhythmic processes, therefore, this result was surprising in that we had hypothesized that chronic stress would decrease or completely disrupt *Npas2* gene expression rhythms. Interestingly, we found that both FSS and UCMS treatment resulted in a dramatic increase in expression of *Npas2*. We propose that these disruptions might contribute to the development of anxiety-like phenotype following acute and chronic stress.

Selective knockdown of *Npas2* in the NAc results in reduced anxiety-like behavior, similar to *Npas2* mutant mice. AAV-mediated knockdown of *Npas2* specifically in the NAc resulted in increased percent time in the open arms of the elevated plus maze, reduced latency to explore the light side of the light/dark box, and a trend for increased distance traveled in the center of the open field arena. NAc *Npas2* knockdown also resulted in a small, but significant difference in locomotor response to novelty, where *Npas2* shRNA treated mice exhibited an initial increased exploratory behavior that decreased more quickly (habituation) than scramble shRNA treated mice. Together, these findings suggest that knocking down NAc expression of *Npas2* recapitulates some of the reduced anxiety-like behaviors seen in the *Npas2* mutant mice. Previous reports, in conjunction with the studies presented here, support a model whereby VTA *Clock* is important for negatively regulating reward and anxiety and NAc *Npas2* is important for positively regulating reward and anxiety (Mukherjee et al., 2010; Coque et al., 2011; Ozburn et al., 2012; Sidor et al., 2015).

We further examined genes that are under the transcriptional control of NPAS2 to identify a mechanism by which NPAS2 could alter behavior. Using ChIP Seq, gene expression and behavioral assays, we found that the direct regulation of *Dopamine receptor 3* (*Drd3*) by NPAS2 in the NAc is important for regulating reward [9]. Genetic manipulation of *Drd3* in mice has not yielded reports of consistent effects on anxiety-like behavior (Steiner et al., 1997; Xing et al., 2013; Moraga-Amaro et al., 2014; Leggio et al., 2015). However, Drd3 antagonism has been shown to reduce (pre-adolescent) stress-induced depression-like behavior in adult mice (Seo and Kuzhikandathil, 2015). Leggio et al. (2015) found that Drd3 KO mice exhibited decreased anxiety-like behavior that was initially more sensitive to the effects of diazepam (as compared to WT mice). To draw comparison, we observe that *Npas2* mutants and NAc *Npas2* KD results in reduced anxiety-like behavior. Further, we observe decreased initial sensitivity to diazepam in Npas2 mutant mice. However, we find that NAc *Npas2* KD results in increased levels of Drd3 (Ozburn et al., 2015). Thus, our findings do not offer insight to the correlations observed in Leggio et al. (2015). The tolerance to diazepam observed in Drd3 KO mice after 3 days of treatment was correlated with increased levels of *Gabra6* (GABAa alpha 6 subunit associated with extrasynaptic receptors and tonic GABA currents; Leggio et al., 2015). We did not measure chronic treatment, so it is difficult to say whether diazepam tolerance would develop differently in *Npas2* mutants. Further, *Gabra6* was not identified via NPAS2 ChIP-Seq, thus we did not measure *Gabra6* levels in mice with NAc *Npas2* KD.

These studies separate the role of NPAS2 regulation of *Drd3* in reward from the role of NPAS2 in measures of anxiety-related behavior and suggest a separate mechanism by which these processes are regulated. Based on our current findings that *Npas2* mutant mice are insensitive to diazepam, we explored the possibility that NPAS2 may act as a positive regulator for the transcription of GABAa subunits. We performed ChIP assays on striatal tissue to isolate DNA bound to NPAS2 and used this DNA as a template for PCR with primers targeting various GABAa subunit genes. We identified that NPAS2 binds genes encoding the GABAa subunits alpha 1, 2, 4 and 5, beta 2 and 3 and gamma 1 and plays an important role in the positive regulation of the GABAa alpha 1 subunit. The binding site for diazepam requires the pentameric subunit composition of the GABAa receptor to contain two alpha subunits (1, 2, 3 or 5) in combination with two beta and one gamma subunit. Thus, the reduced sensitivity to diazepam in *Npas2* mutant mice is likely mediated by a reduction in alpha 1 subunit expression. GABAa alpha1 is thought to be important for the sedative effect of benzodiazepines, whereas alpha 2 and 3 confer the anxiolytic effects. This novel finding indicates that stress increases *Npas2* and NPAS2 mediated transcription of specific GABAa subunits, which may alter inhibitory neurotransmission in the NAc.

Lastly, we tested the functional consequences of *Npas2* knockdown on GABAergic neurotransmission in MSNs of the NAc using *ex vivo* slice electrophysiology. We found that *Npas2* shRNA infected cells had a reduction in mIPSC amplitude, but not frequency. This finding suggests that *Npas2* knockdown may result in postsynaptic modification of MSNs resulting in decreased mIPSC amplitude, but does not appear to affect presynaptic release probability of GABA or the number of functional synaptic sites at MSNs. Further, we found that mice lacking *Npas2* were behaviorally less sensitive to diazepam, and this insensitivity persists at the cellular level in *Npas2* shRNA infected neurons. Diazepam has been shown to potentiate GABAa-receptor mediated currents by binding specific subunit combinations of synaptic GABAa receptors and promoting the binding of GABA, which in turn increases total conductance of chloride (Macdonald and Olsen, 1994; Xu and Sastry, 2005). We found that diazepam application reliably increased the evoked IPSC peak amplitude in scramble control cells by approximately 20% while this increase in current amplitude was noticeably absent in *Npas2* shRNA infected cells. This result suggests that knockdown of *Npas2* in NAc MSNs abolishes the cellular response to diazepam as measured by a change in inhibitory activity. These results provide functional evidence for the regulation of GABAa receptor subunit expression by NPAS2.

We propose that expression of the circadian transcription factor, *Npas2*, is important for stress responses and anxiety-related behaviors and regulates GABAergic inhibitory neurotransmission in MSNs of the NAc. Taken together, these findings support the existence of a homeostatic mechanism by which stress and anxiety increase NPAS2-dependent transcription of specific GABAergic subunits that selectively alter phasic, synaptic inhibitory neurotransmission. Future work will focus on testing the efficacy of pharmacotherapeutics (that target the molecular clock and/or its targets) in ameliorating these adaptations to improve our understanding of and treatments for anxiety-related disorders.

## Ethics Statement

This study was carried out in accordance with the recommendations put forth by the Guide for the Care and Use of Laboratory Animals (9th edition) by the National Research Council (US). All animal use was approved by the University of Texas Southwestern Medical Center, the University of Pittsburgh and the Portland VA Medical Center Institutional Animal Care and Use Committees.

## Author Contributions

ARO, JK, PKP, RWL, ZL, EF, DB-K, KP and NME collected and analyzed data. ARO, JK and PKP prepared the manuscript. ARO, YH and CAM provided resources, guided experimental design and edited the manuscript.

## Supplementary Material

The Supplementary Material for this article can be found online at: https://www.frontiersin.org/articles/10.3389/fnmol.2017.00360/full#supplementary-material

## Conflict of Interest Statement

The authors declare that the research was conducted in the absence of any commercial or financial relationships that could be construed as a potential conflict of interest.
