# Supplementary material for: NPAS2 Regulation of Anxiety-Like Behavior and GABAA Receptors
Source: Front Mol Neurosci. 2017 Nov 3;10:360. doi: 10.3389/fnmol.2017.00360 (PMC5675889; doi:10.3389/fnmol.2017.00360)
Supplement: Supplementary file 1 [file Data_Sheet_1.pdf]

## SUPPLEMENTAL INFORMATION

### Materials and Methods

*Behavioral Assays*

Behavioral testing was carried out between ZT (zeitgeber) 2 and 6. Mice were habituated to testing rooms one hour prior to behavioral testing. Behavioral testing apparatus were cleaned and allowed to dry between mice. Trained, blinded observers scored behaviors.

*Locomotor Response to Novelty*

C57BL/6J mice (tested at UPMC): Mice were individually placed in a novel environment inside automated locomotor activity chambers equipped with infrared photobeams (Kinderscientific Smart Cage Rack System, field dimensions: 9.5" x 18.0") and measurements began immediately [1]. Activity was continuously measured and the data was collected in 5-minute bins over a period of two hours. Distance traveled was measured. An initial exploratory bout of increased activity that decreases over the two-hour testing period is typically seen and represents habituation to the novel environment.

*Elevated Plus Maze*

Npas2 null mutant mice (tested at UTSW): Mice were placed in the center of an elevated plus maze (EPM) (arms are 30x9x5 cm, with 25-cm-high walls on the closed

arms) under low light levels and their behavior was monitored for 5 min [2-4]. The time spent in the closed and open arms, as well as the number of explorations of open and closed arms, were determined by video tracking software (ETHOVISION 3.0; Noldus, Leesburg, VA, USA).

C57BL/6J mice (tested at UPMC): The plus maze consisted of two plastic open arms perpendicular to two closed arms (arms: 30 X 5 cm) and was elevated from the ground at a height of 60cm [1]. Mice were placed into the center of the plus-maze and allowed to explore the maze for 5 min. Behavior was video recorded. Movement and time spent in each arm was automatically tracked (ETHOVISION 3.0; Noldus). More time spent in the open arms, and more open arm entries are both indicative of reduced anxiety-like behavior.

*Light/Dark Box*

Npas2 null mutant mice (tested at UTSW): The dark/light apparatus consisted of two-chambered boxes (25x9x26 cm for each side) (Med Associates, St Albans, VT, USA). One side was kept dark (room light entry limited) and the other side was brightly lit by a fluorescent bulb across the top. Mice were first placed in the dark side for 2 min, then the door between the compartments was opened and they were allowed to freely explore either the light or dark side for 10 min. Anxiety-like behavior was measured as the activity in the light side during the final 10 minutes.

C57BL/6J mice (tested at UPMC): Light/dark test was carried out using the KinderScientific Smart Cage Rack System interfaced to a PC running MotorMonitor

software. Each chamber was equipped with a black Plexiglas box on one side of the chamber; a small doorway allowed free exploration of both dark and light chambers. Mice were initially habituated to the dark chamber for 1 min and then allowed free exploration of both light and dark chambers for the remainder of the 10 min test. Latency to enter and time spent in the light side of the apparatus were measured. Reduced latency to enter the light portion of the apparatus indicates reduced anxiety-like behavior.

*Open Field*

Npas2 null mutant mice (tested at UTSW): Mice were placed in the periphery of a novel open field environment (44x9x44 cm, 30 cm high walls) in a dimly lit room and allowed to explore for 5 min as described previously [2-4]. The animals were monitored from above by a video camera connected to a computer running video tracking software (ETHOVISION 3.0; Noldus) to determine the time, distance moved and number of entries into two areas: the periphery (5 cm from the walls) and center (14x9x14 cm).

C57BL/6J mice (tested at UPMC): The open field consisted of a large 61 cm^3 plexiglass arena with a clear floor and solid black walls [1]. Mice were individually placed into the center of the open field and allowed to explore the arena for 10 min. The center was designated as a 20 cm^2 area in the center of the open field. Behaviors were recorded and scored using Ethovision XT. Anxiety-like behavior was assessed using the following: time spent in the center of the arena and distance traveled in the center. Locomotor activity was measured by total distance traveled. Increased time

spent in the center and distance traveled in the center of the arena indicate reduced anxiety-like behavior.

*Accelerating Rotarod Assay*

Npas2 null mutant mice (tested at UTSW): The rotarod test was performed to evaluate motor coordination. Mice (n=7/genotype) were placed on immobile cylinders, which ramped up from 0 to 45 rotations/min (model 755, IITC Life Science Inc., Woodland Hills, CA). The timer was stopped when the mouse fell off the cylinder or did a whole turn with it. This procedure was repeated for four consecutive days. The first two days consisted of training on the accelerating rotarod. On the last two days, mice were injected with either saline or 3 mg/kg diazepam (in a counterbalanced manner) 30 minutes prior to rotarod testing. For each genotype, latency to fall was compared under diazepam and saline treatment conditions. A reduction in the latency to fall indicates sensitivity to the motor incoordinating effects of diazepam.

*Determination of the effects of acute and chronic anxiogenic stimuli on striatal Npas2 expression: Additional details for Unpredictable Chronic Mild Stress*

C57BL/6J mice (tested at UPMC): Mice were group housed and exposed to six weeks of UCMS or control handling (N=6 mice per group per time point). UCMS treated mice were subjected to a randomized schedule of 1-2 mild stressors per day, seven days per week as described in [5]. Stressors included forced bath (~4cm of water in a rat-sized

cage for 15 min), wet bedding, aversive smell (1h exposure to fox urine), dirty bedding (rotate mice into previously occupied “dirty” cages), tilt cages (45° tilt), restraint (50ml tube for 15 min), reduced cage space, no bedding, and bedding change (replaced soiled bedding with clean bedding). Two or three stressors were intermittently used simultaneously to contribute to the random nature of the paradigm. No light/dark manipulations were used. Fur rating and body weights were measured weekly to track the progression of the “UCMS syndrome”. Control animals were housed in the same room as the UCMS exposed animals and only handled for fur ratings and body weight measurements. To ensure UCMS treated mice exhibited increased anxiety-like behaviors associated with UCMS syndrome, mice were subjected to elevated plus maze and light/dark box testing (on two separate days) during the 5$^{th}$ week of UCMS, and thus did not receive randomized stressors on these days. The behavioral data accompanying this experiment is part of another publication focusing on the effects of UCMS on circadian gene expression in the SCN [5]. Stressors were purposely conducted at random times throughout the day to avoid potential non-specific circadian effects of acute stressors. Immediately after 6 weeks of UCMS or control handling, mice were sacrificed by cervical dislocation and rapid decapitation. Whole brains were dissected and flash frozen on dry ice, sectioned on a cryostat at 200um, and NAc tissue was collected using a 1mm core tissue puncher.

*Quantitative Real-Time RT-PCR*

For FSS tissue: Total RNA was extracted using Trizol (Life Techologies, Grand Island, NY) and converted to cDNA using the Superscript III First Strand Synthesis Kit (Life Technologies, Grand Island, NY). cDNA was mixed with SYBR Green master mix

(Applied Biosystems, ABI) and specific primers for *Npas2* or *Gapdh* (primer sequences, mastermix, and qPCR machine listed below). Prior to the experiment primer sets were tested thoroughly to determine reaction efficiency, specificity, and the absence of primer-dimers [6]. Minimum Information for Publication of Quantitative Real-Time PCR Experiments, or MIQE guidelines were adhered to for all data presented here and are described in [7, 8].

For UCMS tissue: Total RNA was extracted using an RNeasy Micro Kit (Qiagen, Germantown, MD) and converted to cDNA using the Superscript III First Strand Synthesis Kit (Life Technologies, Grand Island, NY). cDNA was mixed with SYBR Green master mix (Applied Biosystems, ABI) and specific primers for *Npas2* or *Gapdh*. Primer sequences for *Npas2* were (forward) 5'-GACACTGGAGTCCAGACGCAA-3' and (reverse) 5'-AATGTATACAGGGTGCGCCAAA-3' and for *Gapdh* (forward) 5'-AACGACCCCTTCATTGAC-3' and (reverse) 5'-TCCACGACATACTCAGCAC-3'. Quantitative real-time RT-PCR reactions were assessed by SYBR green fluorescence signal (Power SYBR Green PCR Master Mix, Life Technologies) using a 7900HT Fast Real-Time PCR System (Applied Biosystems, Grand Island, NY).

For *Npas2* shRNA and Scramble shRNA transduced NAc tissue: Tissue was collected, RNA was isolated, and cDNA synthesis was performed and is described in Ozburn and Falcon et al., 2015. Because only a small amount of RNA was isolated from these samples, we moved forward with the BioRad multiplex fluorophore PCR system to measure relative gene expression of multiple gamma-aminobutyric acid receptor (type A) subunits of interest (and housekeeping gene Gapdh) in the same sample. In brief, cDNA was mixed with SsoAdvanced™ Universal Probes Supermix (Bio-Rad

Laboratories; Hercules, CA) and all five probes for genes of interest (listed in Supplementary Table 1). Reactions were carried out in a Bio-Rad CFX96 Touch™ Real-Time PCR Detection System using the Bio-Rad CFX Manager 3.1 software (Bio-rad Laboratories). Scan mode was set to 'all channels' to allow for detection of each of the five fluorophore reporters.

**Supplementary Table 1**

| **PrimePCR Probes, Mouse (Bio-Rad)** | | | |
|---|---|---|---|
| **Gene** | **Gene Name** | **Reporter** | **Cat#** |
| *Gapdh* | Glyceraldehyde 3-phosphate dehydrogenase | Cy5.5 | 10031237 |
| *Gabrg1* | Gamma-Aminobutyric Acid Type A Receptor Gamma1 Subunit | FAM | 10031228 |
| *Gabrg2* | Gamma-Aminobutyric Acid Type A Receptor Gamma 2 Subunit | HEX | 10031231 |
| *Gabra1* | Gamma-Aminobutyric Acid Type A Receptor Alpha 1 Subunit | TEX 615 | 10031234 |
| *Gabra2* | Gamma-Aminobutyric Acid Type A Receptor Alpha 2 Subunit | Cy5 | 12001950 |

For all reactions: Samples were run in duplicate and ΔCT values were determined by normalizing to the reference gene *Gapdh*. Relative expression values were calculated based on the following: $\Delta\Delta CT=(\Delta CT_{\text{highest value across groups}} - \Delta CT_{\text{sample}})$ then relative expression $((2^{-\Delta\Delta CT}{}_{\text{sample}} / 2^{-\Delta\Delta CT}{}_{\text{highest value across groups}})*100)$ was calculated as described in [2].

*Stereotaxic surgery*

Stereotaxic surgery was performed as described [6]. Mice were anesthetized with a mixture of ketamine (100 mg/kg) and xylazine (10 mg/kg) in saline (0.9% NaCl). Bilateral stereotaxic injections of 1 ul of purified high titer AAV2 encoding a scrambled sequence with no known target (scramble shRNA) or *Npas2* shRNA was injected into the NAc (from bregma: angle 10°, AP +1.5 mm, Lat +1.5mm, DV -4.4mm) using a 33 gauge hamilton syringe (Hamilton, Reno, NV). Plasmids were sent to the University of North Carolina Gene Therapy Viral Vector Core for packaging into adeno-associated viral (AAV2) vectors (Chapel Hill, NC). The viral constructs also express green fluorescent protein (GFP) to aid in verification of injection placement. Injection rate was 0.1 µl/minute, and the needle was kept in place for an additional 5 minutes before it was slowly withdrawn. Mice recovered for four weeks in their home cage to allow for full viral expression before behavioral testing began (n=10-13/treatment).

*Immunohistochemical localization of AAV expression*

Mice were deeply anesthetized with a mixture of ketamine (225mg/kg) and xylazine (22.5mg/kg) and transcardially perfused with phosphate buffered saline (PBS) followed by 4% paraformaldehyde in PBS. The brains were incubated in 4% paraformaldehyde for 24 hours and then placed in 1X PBS-30% glycerol for an additional 24 hours. Tissue sections (30 um) containing the NAc were obtained using a freezing microtome (Leica, Wetzlar, Germany). Immunofluorescence detection was carried out using a primary rabbit anti-GFP antibody (ab290, AbCam, Cambridge, MA) and a secondary anti-rabbit antibody conjugated with Alexa-488 (Molecular Probes, Carlsbad, CA) using standard procedures [3, 6, 9]. Brain sections were mounted onto glass slides using Vectashield mounting media with DAPI (H-1200, Vector Labs, Burlingame, CA) and observed with

an epifluorescence microscope. Data from mice were excluded from analysis if the viral expression of GFP was low, spread was not localized to the NAc (with spillover to adjacent areas) or if there was a significantly disproportionate amount of infection between both hemispheres.

*Chromatin Immunoprecipitation*

Chromatin immunoprecipitation was carried out as described in [6]. Ozburn and Falcon et al. (2015) performed ChIP Seq at 6 times of day and identified novel DNA binding targets of NPAS2. Here, we performed a separate ChIP experiment using an NPAS2 antibody (H20X, Santa Cruz Biotechnology, Santa Cruz, CA) to confirm findings that NPAS2 binds to several genes encoding subunits of the GABAA receptor. As a control, we also incubated samples with Anti-acetyl-Histone H3 (Upstate) or non-immune rabbit IgG (Upstate). PCR products were visualized and size verified using agarose gel electrophoresis. Primer sets used for PCR verification of ChIP Seq results include: *Gabra1* Forward 5' GCT CTA AAA GCT GGA GAG TAG CAC C, Reverse 5' CCC AGT CCT TCT TTA TAG GCA CCG C; *Gabra2* Forward 5' TGG GAA GAT TGT AAC CCG TCC CCC, Reverse 5' CCT GTC ATA GCC CTG TGA GCC ACC; *Gabra4* Forward 5' GCC CTG CTT CCA CAG CAA CAC AC, Reverse 5' GCC AAA TAC CTG GCC TCA GCA GC; *Gabra5* Forward 5' CCC AGA CAA GCA AGG GCT GAC CC, Reverse 5' AGC CCA AGG AGA GTC CAG ACT GAT T; *Gabrb1* Forward 5' ACT GCA CAG CAC AGT GAG AGA GAG T, Reverse 5' ACA CAC ACA CTC ACA CAC ACA CAG A; *Gabrb2* Forward 5' ATC ACT GAC TGC TAG GAT GCG ACT, Reverse 5' GAG TCC TAT TGC CCG ATG CAA GGC; *Gabrb3* Forward 5' GGG AGG AGA GTG TAT TGT CCT GGT, Reverse 5' ACA GTG CTA ACG GAG CAG AGC CA; *Gabre* Forward 5'

GGG CTC TGA TTT CAT CTC TGG CTC, Reverse 5’ AAC CGG AGC CCC ATC CCC A; *Gabrg1* (SET 1) Forward 5’ AAA AGG CAT GCA CAT GGT TGG GTG A, Reverse 5’ CCT CAG CTG CAT CCC TGA CCC TC; and *Gabrg1* (SET 2) Forward 5’ TCC CTC GGG AAC CCG ACT CTC A, Reverse 5’ TCA CAC CGG GTG GAT GCG GC.

*Slice Electrophysiology*

Three weeks after stereotaxic injection of AAV *Npas2* or scramble shRNA into the NAc, mice were rapidly anesthetized with isoflurane and decapitated. Brains were removed into ice-cold oxygenated (95% $O_2$/5% $CO_2$) modified aCSF containing (in mM): 135 *N*-methyl-D-glucamine, 1 KCl, 1.2 $KH_2PO_4$, 1.5 $MgCl_2$, 0.5 $CaCl_2$, 70 choline bicarbonate, and 10 D-glucose; pH 7.4 adjusted with HCL; pH 7.4 adjusted with HCl). Coronal slices (250 um) containing the NAc were sectioned with a vibratome (VT1200S; Leica) and incubated for 30 minutes at 37°C in oxygenated aCSF containing (in mM): 119 NaCl, 26 $NaHCO_3$, 2.5 KCl, 1 $NaH_2PO_4$, 2.5 $CaCl_2$, 1.3 $MgCl_2$, 11 D-glucose. Slices were kept at room temperature until recording at which point they were perfused with aCSF heated to 30-32°C. Whole-cell patch-clamp recordings of identified MSNs with viral expression (as indicated by GFP fluorescence) were made under visual guidance with 40x objective and DIC optics. Borosilicate glass pipettes (3-5 MΩ) were filled with (in mM): 15 Cs-$MeSO_3$, 120 CsCl, 10 HEPES, 0.5 EGTA, 8 NaCl, 5 TEA-Cl, 2 Mg-ATP, 0.3 Na-GTP, 5 QX-314; 290mOsm; pH 7.3 adjusted with CsOH. For miniature IPSC (mIPSC) and evoked IPSC experiments, D-APV (50 uM) and NBQX (5 uM) were included to block ionotropic glutamate receptors and TTX (1 μM) was used to prevent action potential generation in mIPSC recordings. Drugs were bath applied. Cells were voltage-

clamped at -70 mV and held for approximately 10 minutes prior to data collection. A constant-current isolated stimulator (DS3; Digitimer) was used to stimulate inhibitory afferents through a monopolar electrode at 0.1 Hz using 0.1 μs single pulses. After establishing a stable baseline of mIPSCs or IPSCs, 10 μM diazepam-containing aCSF was bath applied at a consistent flow rate over 10 minutes. Synaptic currents were recorded with a MultiClamp 700B amplifier (Molecular Devices, Sunnyvale, CA). Signals were filtered at 2.6-3 kHz and amplified 5-10 times, then digitized at 20 kHz with a Digidata 1322A analog-to-digital converter (Molecular Devices). Series resistance for all recordings was <20MΩ and was monitored continuously. Cells with a change in series resistance beyond 20% were excluded from data analysis. For mIPSC analysis, an event template was obtained by averaging at least 50 single events and used for template search with a threshold of 4. Events from each cell underwent visual inspections. Scoring was performed blind to treatment. The amplitude and frequency of miniature events were analyzed offline with Clampfit 10.3 software (Molecular Devices). Peak amplitude of evoked IPSCs was measured and averaged across baseline and treatment conditions. All data is presented as mean +/- S.E.M., with n/n representing the number of cells/animals.

**Supplemental Figure 1.**

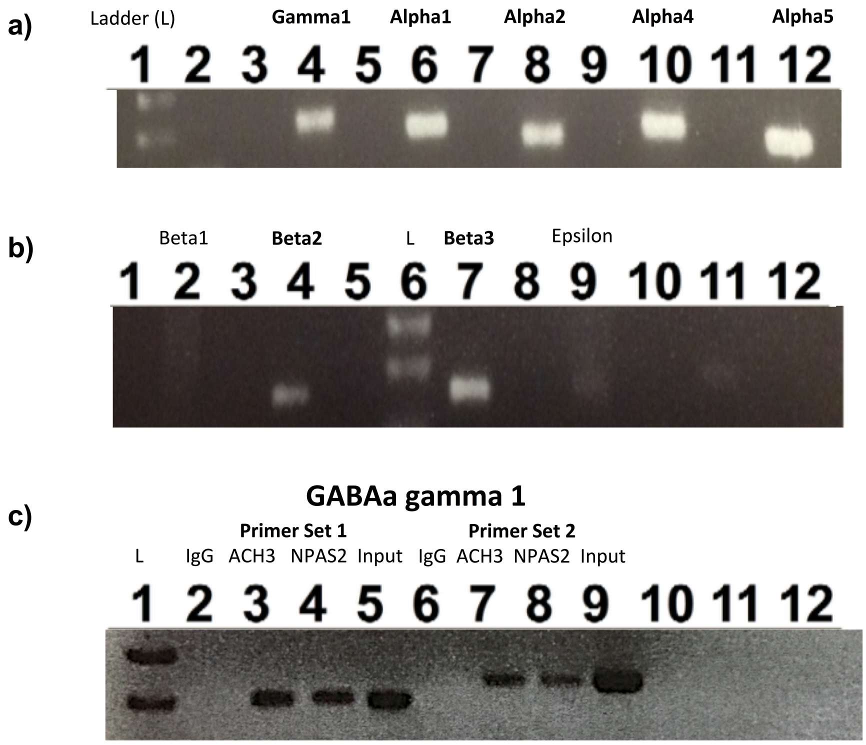


**S1.** NPAS2 binds many novel gene targets, including GABAA subunits alpha 1, 2, 4, and 5, beta 2 and 3, and gamma 1. ChIP assays followed by PCR and gel analysis A) NPAS2 binds to *Gabrg1*, *Gabra1, GAbra2, Gabra4, Gabra5* genes, B) NPAS2 does not bind to *Gabrb1* or *Gabre* genes, but does bind to *Gabrb2* and *Gabra3* genes, C) NPAS2 binds to 2 distinct regions of the *Gabrg1* gene that is also bound by acetylated histone H3 but not IgG (negative control for ChIP).

## References


1. Sidor, M.M., et al., *Daytime spikes in dopaminergic activity drive rapid mood-cycling in mice.* Mol Psychiatry, 2015. **20**(11): p. 1479-80.
2. Spencer, S., et al., *Circadian genes Period 1 and Period 2 in the nucleus accumbens regulate anxiety-related behavior.* Eur J Neurosci, 2013. **37**(2): p. 242-50.
3. Mukherjee, S., et al., *Knockdown of Clock in the ventral tegmental area through RNA interference results in a mixed state of mania and depression-like behavior.* Biol Psychiatry, 2010. **68**(6): p. 503-11.

4. Roybal, K., et al., *Mania-like behavior induced by disruption of CLOCK.* Proc Natl Acad Sci U S A, 2007. **104**(15): p. 6406-11.
5. Logan, R.W., et al., *Chronic Stress Induces Brain Region-Specific Alterations of Molecular Rhythms that Correlate with Depression-like Behavior in Mice.* Biol Psychiatry, 2015. **78**(4): p. 249-58.
6. Ozburn, A.R., et al., *Direct regulation of diurnal Drd3 expression and cocaine reward by NPAS2.* Biol Psychiatry, 2015. **77**(5): p. 425-33.
7. Ozburn, A.R., et al., *Functional Implications of the CLOCK 3111T/C Single-Nucleotide Polymorphism.* Front Psychiatry, 2016. **7**: p. 67.
8. Bustin, S.A., et al., *The MIQE guidelines: minimum information for publication of quantitative real-time PCR experiments.* Clin Chem, 2009. **55**(4): p. 611-22.
9. Ozburn, A.R., et al., *The role of clock in ethanol-related behaviors.* Neuropsychopharmacology, 2013. **38**(12): p. 2393-400.
